# Multi-Response Optimization of Ultrasonic Assisted Enzymatic Extraction Followed by Macroporous Resin Purification for Maximal Recovery of Flavonoids and Ginkgolides from Waste *Ginkgo biloba* Fallen Leaves

**DOI:** 10.3390/molecules23051029

**Published:** 2018-04-27

**Authors:** Guisheng Zhou, Jiayan Ma, Yuping Tang, Xinmin Wang, Jing Zhang, Jin-Ao Duan

**Affiliations:** 1 Jiangsu Collaborative Innovation Center of Chinese Medicinal Resources Industrialization, Nanjing University of Chinese Medicine, Nanjing 210023, China; zhouguisheng1@126.com (G.Z.); 18762852628@163.com (J.M.); ZJ199303@126.com (J.Z.); 2 College of Pharmacy and Shaanxi Collaborative Innovation Center of Chinese Medicinal Resources Industrialization, Shaanxi University of Chinese Medicine, Xi’an 712046, China; 3 Center for ADR monitoring of Jiangsu, Nanjing 210002, China; yatou8880@126.com

**Keywords:** *Ginkgo biloba* fallen leaves, ultrasonic–assisted enzymatic extraction, Plackett–Burman design, response surface methodology, macroporous resin

## Abstract

In the present study, the process of ultrasonic assisted enzymatic extraction (UAEE), followed by macroporous resin purification, was successfully developed to achieve maximal recovery of flavonoids and ginkgolides from *Ginkgo biloba* fallen leaves (GBFL). Three effective extracted factors, including UAE power, EtOH%, and the amount of cellulase were screened by Plackett–Burman design (PBD). The important variables were further optimized by rotatable central composite design (RCCD). After the combination of PBD and RCCD, the resulting optimal UAEE conditions were as follows: UAE power of 218 W; EtOH% of 68%; the amount of cellulase of 8.4 mg; UAE temperature of 40 °C; UAE time of 20 min; pH of 5.0; and, sample particle size of 40 mesh. Under the optimum conditions; the yields of flavonoids were 0.74 ± 0.05% (*n* = 3) and ginkgolides was 0.42 ± 0.06% (*n* = 3), which were close to the predicted values. Moreover, the further enriching flavonoids and ginkgolides from the obtained GBFL extracts using the above optimum UAEE condition was successfully achieved by macroporous resin DA-201. After column adsorption and desorption on DA-201; the percentage of total flavonoids was (25.36 ± 1.03)%; ginkgolides was (12.43 ± 0.85)% and alkylphenols was (0.003 ± 0.0005)% from the obtained dry extracts of GBFL which were complied with Chinese pharmacopoeias. Therefore, the present study provided a convenient and efficient method for extraction and purification of flavonoids and ginkgolides from waste GBFL.

## 1. Introduction

*Ginkgo biloba* L. (*G. biloba*), the only remaining member of the Ginkgoaceae family, was widely distributed in extratropical, warm and subtropical zones, especially in eastern China. *G. biloba* has existed on the earth for 200 million years, but its true value has induced a range of attentions all around the world until the recent five decades. The extract from *G. biloba* leaves (EGb) was among the most commonly used herbal medicines and/or dietary supplements. There was an increasing evidence of the potential role of GBE to treat neurodegenerative diseases [1,2], intermittent claudication [3], tinnitus [4,5], and many other diseases. Nowadays, *G. biloba* leaves was one of the most sold medicinal plants of estimates of worldwide annual sales, and EGb was standardized for the two fractions of main active ingredients [6], namely flavonoids and ginkgolides. EGb has been of great interest to the food and pharmaceutical industries due to their inimitable benefits [7].

China was the home country of *G. biloba* and possessed more than 70% of the world's total of the ginkgo resources. The standardized EGb were prepared from *G. biloba* leaves that were collected in July and August from four to seven-year-old trees (for the convenient description, the *G. biloba* leaves collected in July and August from four to seven-year-old trees were abbreviated as “GBL”), due to the relative high contents of flavonoids and ginkgolides in GBL. GBL was paid more attention on most research, however a large number of fallen leaves from fruit cultivars (tree older than 10 years) were ignored and become obsolete after the fruit harvest season (November). *G. biloba* fallen leaves (GBFL) was treated as waste to incinerate and/or discarded abundantly in soil and water, thus it polluted air, soil and, rivers. Additionally, GBFL have become an urgent problem to the local enterprises and governments of the planting area for its poison to the environment. This waste GBFL were not presently being utilized for any value-added processes due to limited research activities focusing on the possible conversion of the waste to other valuable products, thereby making GBFL available for dumping as waste. Therefore, the development of new, environment-friendly method to manage that problem was great concern. From the previous reports and our researches, the total contents of flavonoids and ginkgolides from GBFL were about 30% and 40% in GBL, respectively. Additionally, with the increasing requirement of EGb, the resources were in shortage for GBL. So, the conversions of waste GBFL into valuable by product, such as flavonoids and ginkgolides, might offer a great scope for its utilization, lessened the pressure of GBL resources, created more income for farmers, and more importantly, reduced environmental impacts of the waste.

Extraction of flavonoids and ginkgolides might be an efficient strategy to solve the problem of utilization of waste GBFL. Extraction techniques used for different compounds were distillation, solvent extraction (maceration, digestion, infusion, decoction, percolation, and hot continuous extraction), countercurrent extraction, cold compression, and non-conventional methods, namely supercritical fluid extraction (SFE), microwave-assisted extraction (MAE), ultrasound-assisted extraction (UAE), and ultrasonic assisted enzymatic extraction (UAEE) [8]. When compared with conventional extraction methods, UAEE was a simple, rapid, inexpensive, and efficient method for extracting target compounds from plant material. From the previous reports, UAE exhibited the best mass transfer, cell disruption, solvent penetration, and capillary effect, and it was the most economical technique to scale up for industrial production [9]. Additionally, the release of intracellular contents was also promoted by breaking the cell wall when enzymes, such as cellulases and proteases, were added in the solution of UAE [10,11,12]. So, the combinational usage of UAE and enzyme would be more effective probably during the extraction of the target compounds. In the process of UAEE, many factors, including ultrasound power, extraction time and temperature, cellulase concentration, pH and solvent-to-material ratio, etc., could influence the extraction process, individually and collectively. Therefore, the optimization of the extraction parameters was required to obtain the maximum yield of flavonoids and ginkgolides from waste GBFL. As a mathematical and statistical technique, Plackett-Burman designs (PBDs), combined with the response surface method (RSM) was a useful method for evaluating multiple parameters and their interactions based on quantitative data and might effectively overcome the drawback of classic “one-factor-at-a-time” or “full-factors” approach [13]. Additionally, PBDs-RSM was frequently used to optimize the extraction parameters of target compounds from different plant materials.

In the process of enrichment, macroporous resin, which is an organic polymer adsorbent, has been widely used to adsorb and enrich target compounds in medicine, food, chemistry, and other fields owing to its high adsorption and desorption capacity, low cost, and simple equipment [14]. Furthermore, the use of these resins were easily regenerated and not affected by the presence of inorganic materials [15]. Therefore, in this study, macroporous resin was employed to further enrich flavonoids and ginkgolides.

The objective of this study was to develop a process to optimize the recovery of flavonoids and ginkgolides from waste GBFL. First, UAEE parameters were screened by PBD, and the operational parameters of the extraction were optimized by RSM. Then, the purification was performed by macroporous resin chromatography. This study reported the effect of extraction and purification of flavonoids and ginkgolides from waste GBFL for the first time, which will provide a scientific basis for the comprehensive utilization and development of *G. biloba* resources.

## 2. Results and Discussion

### 2.1. Identifying and Profiling of Flavonoids and Ginkgolides from GBFL Extracts

It was necessary to develop a simple, rapid, and accurate analytical method to the identify and profile flavonoids and ginkgolides in different matrices of GBFL. Based on several previously reported HPLC-DAD and HPLC-MS methods, we developed an ultra-high performance liquid chromatography coupled with diode array detector-Q/TOF mass spectrometry (UPLC-DAD-Q/TOF-MS) method for the identification and the determination of flavonoids and ginkgolides in GBFL.

#### 2.1.1. Optimization of UPLC-DAD-Q/TOF-MS Conditions

In the preliminary studies, stationary phase, mobile phase, and other instrument parameters were optimized to obtain ideal UPLC elution conditions for the simultaneous separation of target compounds. A mixed solution of 26 (20 flavonoids, four ginkgolides, and two alkylphenols) standards and the crude extract of GBFL were employed to optimize UPLC conditions.

Three candidate columns with different particle sizes and lengths, (1) Acquity UPLC BEH C_18_ column (2.1 × 50 mm, 1.7 μm); (2) Acquity UPLC BEH C_18_ (2.1 × 100 mm, 1.7 μm); and, (3) Acquity HSS T3 (2.1 × 100 mm, 1.7 μm), were compared in a pilot test to achieve a complete separation. It was found that column 2 showed better performance than columns 1 and 3 in terms of capacity, retention ability, and sensitivity for both standards and the crude extract. Additionally, the results of the optimization of the mobile phase showed that acetonitrile/0.1% formic acid water mobile phase system had better resolution and shorter duration of analysis and could improve the peak symmetry and the ionization of the most analytes. Other parameters, such as the flow rate, injection volume and column temperature, were also optimized to obtain a fast and reliable separation. The optimized values were listed in Section 4.2.

Regarding MS conditions, both ESI^−^ and ESI^+^, were investigated. ESI^−^ provided higher sensitivity, cleaner mass spectra and lower background for flavonoids and ginkgolides, which was in agreement with our previously reported results [16]. Hence, ESI^−^ was adopted in this study. Additionally, the other parameters of MS were following as our previous report [16].

#### 2.1.2. Identifying and Profiling of Flavonoids and Ginkgolides

Using the optimal UPLC–DAD–Q/TOF–MS, 26 components were unambiguously identified in GBFL extracts by comparing their retention times, UV spectra, the accurate masses, and fragment ions with those of respective standards. The detailed MS information of all the identified compounds are shown in Table 1.

In negative ion modes, flavone aglycones mainly gain fragment ions by the reverse Diels-Alder (RDA) reaction and the loss of CO (28 Da). The characteristic fragmentation behavior of compound **3** is listed in Table 1. Compound **3** presented [M−H]^−^ at *m*/*z* 285.0558 (C_15_H_9_O_6_), and the characteristic ions were at *m*/*z* 255.0182 [M−H−CH_2_O]^−^, 227.0515 [M−H−CH_2_O−CO]^−^, and 151.0035 [M−H−C_8_H_6_O_2_]^−^. Therefore, compound **3** was identified as kaempferol by comparing with the authentic standard. Using the similar method, the rest 13 flavone aglycones, including compounds **1** (quercetin), **2** (apigenin), **4** (isorhamnetin), **5** (genkwanin), **6** (amentoflavone), **7** (bilobetin), **8** (ginkgetin), **9** (isoginkgetin), **10** (sciadopitysin), **11** ((−)-epigallocatechin), **12** ((+)-catechin hydrate), **13** ((−)-epicatechin), and **14** (luteolin) were unambiguously characterized by comparing with standards. Flavone glycosides have the similar fragmentation pathways of simultaneous or successive losing glucose (162 Da) and/or rhamnose (146 Da). Compound **15** presented [M−H]^−^ ion at *m/z* 739.2543 (C_33_H_40_O_19_). It further generated product ions at *m*/*z* 593.1370, 447.0830, and 285.0626 through consecutive neutral losses of coumaroyl (146 Da), rhamnosyl (Rha-, 146 Da), and glucosyl (Glc-, 162 Da) moieties, respectively. The aglycone ion at *m*/*z* 285.0626 could be from successive or simultaneous losses of CO (28 Da), CH_2_O (30 Da) and C_8_H_6_O_2_ (134 Da) and reversed RDA reaction. Therefore, compound **15** was identified as kaempferol 3-*O*-α-*l*-[6‴-p-coumaroyl-(β-*d*)-glucopyranosyl-(1,2)-rhamnopyranoside] by comparing with the authentic standard. Using the similar method, the rest five flavone glycosides, including compounds **16** (quercetin 3-*O*-α-*l*-[6‴-p-coumaroyl-(β-*d*)-glucopyranosyl-(1,2)- rhamnopyranoside]), **17** (quercetin 3-*O*-[6-*O*-(α-*l*-rhamnosyl)-β-*d*-glucoside]), **18** (quercetin 3-*O*-β-*d*-glucoside), **19** (quercetin 3-*O*-[4-*O*-(α-*l*-rhamnosyl)-β-*d*-glucoside]), and **20** (quercetin 3-*O*-α-*l*-rhamnoside) were unambiguously characterized by comparing with standards.

The [M−H]^−^ ions of bilobalide and ginkgolides A, B, and C were listed in Table 1. Ginkgo terpene lactones exhibited similar fragmentation pathways, with bilobalide differing slightly from the other ginkgolides. Terpene lactones possessed unique chemical structures and were highly oxidised terpenes with many carbonyl and hydroxyl functional groups. Generally (although differences occur across the ginkgolides), loss of single and multiple H_2_O, CO, CO_2_ molecules from [M−H]^−^ was observed, as described by other authors [17,18]. Using the optimal UPLC-DAD-Q/TOF-MS, compounds **21** (bilobalide), **22** (ginkgolide A), **23** (ginkgolide B), and **24** (ginkgolide C) were unambiguously characterized by comparing with the standards.

For quantitative detection, an extract ion chromatogram (EIC) of the full scan MS experiment was validated and employed to assess the quantity of the identified 20 flavonoids (compounds **1**–**20**) and 4 ginkgolides (compounds **21**–**24**) in different matrices of GBFL. The total contents of flavonoids (TF) and ginkgolides (TG) were the sum of identifying 20 flavonoids (compounds **1**–**20**) and 4 ginkgolides (compounds **21**–**24**), respectively. Additionally, the toxic alkylphenols, including ginkgoneolic acid (**25**) and ginkgolic acid (**26**) in different matrices of GBFL, were also identified and investigated by UPLC-DAD-Q/TOF-MS, as described by the previous report [19].

### 2.2. Screening of Significant Factors Using PBD

**Figure 1:**
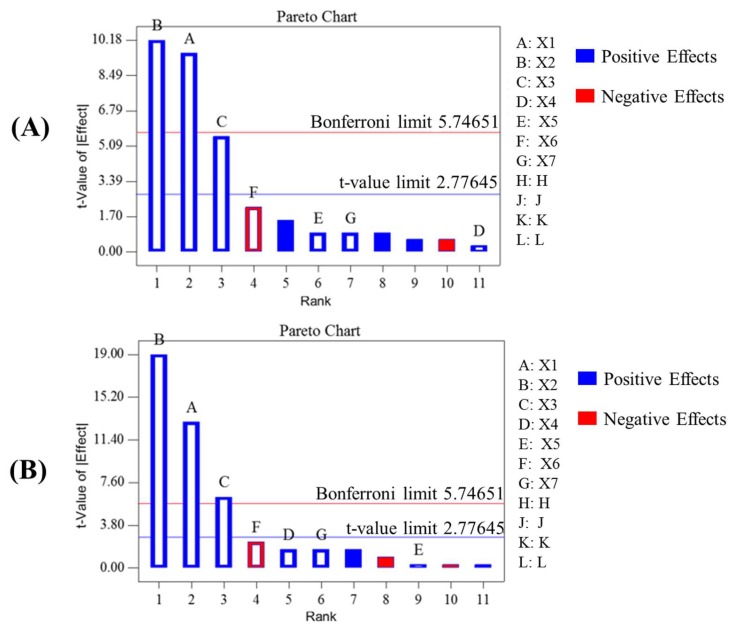
Pareto chart showing evaluated seven variables on the yields of flavonoids (**A**) and ginkgolides (**B**) from *G. biloba* fallen leaves. Variables with t-values higher than the critical value (2.776) were regarded as being statistically significant.

A simple and useful PBD was selected as a method of screening design by a relatively few experiments in this study. Based on the preliminary tests and previous reports on the UAEE, seven variables, including UAE power (A), EtOH% (B), the amount of cellulase (C), UAE time (D), UAE temperature (E), pH (F), and GBFL particle size (G) were determined and are listed in Table 2. The experiment design matrix with Y_1_ (the yield of TF) and Y_2_ (the yield of TG) as responses were also listed in Table 2, and the result and analysis of variance (ANOVA) were presented in Table 3. In general, variables of a large coefficient with a small *p*-value (<0.05) were considered as a significant influence. The results indicated that UAE power (A), EtOH% (B) and the amount of cellulase (C) were considered as significant for two responses (Y_1_ and Y_2_). From the previous reports, pareto chart could present the effect of factors on responses and check the statistical significance. In the pareto chart, two limit lines including Bonferroni limit line (5.746) and *t*-value limit line (2.776) were used to determine the extremely significant, significant, and insignificant coefficients of different factors when *t*-value of effect above the Bonferroni limit line, between the Bonferroni limit line and *t*-value limit line, and below *t*-value limit line, respectively [20]. Additionally, Pareto charts confirmed that the positive or negative sign (in blue or red) response can be enhanced or reduced, respectively, when passing from the lowest to the highest level set for the specific factor [21]. Thus, the pareto chart could intuitively provide significant factors and it was employed to identify the significant factors in this study. Figure 1 showed the result of pareto chart plotted by the *t*-value of effect versus each parameter. As shown in Figure 1B, the *t*-value of UAE power (A) and EtOH% (B) were above Bonferroni limit line, and the *t*-value of the amount of cellulase (C) was between the Bonferroni limit line and *t*-value limit line, both of which indicated that the three variables were considered as significant factors for the yield of TF (Y_1_). The result in Figure 1B indicated that the *t*-value of UAE power (A), EtOH% (B), and the amount of cellulase (C) were above Bonferroni limit line, it exhibited that the above three factors were the extremely significant factors to the yield of TG (Y_2_). The R^2^ and Adj-R^2^ values of two responses (Y_1_ and Y_2_) were more than 95%. The result indicated that the description of pareto charts were dependable. The initial first order model equations developed by PBD for Y_1_ and Y_2_ was generated by the Design-Expert 8.5 software, according to the following equation:

(1) $$\Upsilon_{1\;}=0.014\;+\;0.0005A\;+\;0.004B\;–\;0.01C\;+\;0.0002D\;+\;0.0002E\;–\;0.008F\;+\;0.0002G$$

(2) $$\Upsilon_{2}\;=\;-0.102\;+\;0.0003A\;+\;0.003B\;+\;0.005C\;+\;0.0004D\;+\;0.00003E\;–\;0.004F\;+\;0.0002G$$

where Υ_1_, Υ_2_, A, B, C, D, E, F, and G represented the yield of TF, the yield of TG, the values of UAE power, EtOH%, the amount of cellulase, UAE time, UAE temperature, pH, and GBFL particle size, respectively.

From the final results of PBD, UAE power (A), EtOH% (B), and the amount of cellulase (C) were comprehensively considered as three important factors for further RCCD experiments of maximum the yields of TF (Y_1_) and TG (Y_2_), whereas the remaining four parameters, including UAE time, UAE temperature, pH, and GBFL particle size were regarded as non-significant variables. In the present study, the effects of four non-significant factors were also investigated in the preliminary experiments. The results indicated that a relatively long UAE time might help to increase the contact chance between GBFL and extraction solvent, and raw materials with small particle size contributed to enlarge contact area between powders and extraction solvent. Therefore, longer UAE time and smaller particle size might contribute to enhance the yields of TF and TG. Additionally, the appropriate rise of extraction temperature was advantageous to the molecular motion, the penetration of solvent and dissolution of target compounds. In this study, cellulases were added in the solution of UAE to break the cell wall for promoting the release of intracellular target compounds. From the previous reports, cellulases, a kind of important hydrolase, were influenced by many factors such as temperature and pH. Hence, the appropriate value of pH and temperature can promote to maintain the cellulases activity. For saving cost and improving efficiency, comprehensive consideration, the conditions were selected as following: UAE temperature of 40 °C, UAE time of 20 min, pH of 5.0, and sample particle size of 40 mesh.

### 2.3. Parameter Optimization by RCCD

#### 2.3.1. Statistical Analysis and Model Building

The significant factors that were chosen from UAE power (A), EtOH% (B) and the amount of cellulase (C) were considered for further optimization using RCCD. The levels chosen for the factors were set based on the previous PBD analysis. The RCCD with α = 1.68 has been carried out on 20 experimental runs (2^3^ + (2 × 3) + 6), including eight (2^3^) vertex points, six (2 × 3) axis points, and six center points. Multiple regression analysis was performed on the experimental data (Table 4) to evaluate for significance. The statistical significance of each coefficient of regression equation was checked by *F*-value and *p*-value, thus in turn, indicating the interactions of the variables. Generally, model with very small *p*-value (*p* < 0.05) indicated that the model term was significant, meanwhile, the larger *F*-value and lower *p*-value indicate the significance of each term. ANOVA was performed to retain the significant terms (*p* < 0.05) and exclude the non-significant terms (*p* > 0.05). The result indicated that A, B, C, A^2^, B^2^, and E^2^ were significant for the yield of TF, and A, B, C, AB, A^2^, B^2^, and E^2^ were significant for the yield of TG.

In this study, the models were evidenced to be significant for two responses (the yields of TF and TG) by the large *F*-value (46.61 and 82.03, respectively) and the low *p*-value (<0.0001 and <0.0001, respectively). The “Lack of fit” test was employed to measure the failure of the model to fit the experiment data. The *p*-values of “Lack of fit” for the yields of TF and TG were 0.0763 and 0.7323, respectively. These results indicated that the models could excellently fit the experimental values and predict the response variable.

High R^2^ of the quadratic regression model indicated that the model was workable. The results demonstrated that the proposed regression model for the yield of TG was satisfactory with high R^2^ (0.9866), Adj-R^2^ (0.9746) and pre-R^2^ (0.9513) values, respectively, which exhibited a close agreement between the experimental and the theoretical values. Similar results were also obtained for the yield of TF with high R^2^ (0.9767), Adj-R^2^ (0.9558), and pre-R^2^ (0.8517) values. Meanwhile, the very low coefficient of variances (CV) of the yields of TF and TG were 5.67 and 5.02, respectively, which clearly indicated the very high degree of precision and the great deal of reliability of the experimental values. The detail results of multiple regression analysis were listed in Table 4. By applying multiple regression analysis on the experimental data, the simplified models for the yields of TF and TG was generated by the Design–Expert 8.5 software, according to the following equation:

(3) $$\Upsilon_{\left(\mathrm{TF}\right)}=–2.311\;+\;0.004A\;+\;0.065B\;+\;0.083C\;–\;{0.000008A}^{2}\;–\;{0.0005B}^{2}\;–\;{0.005C}^{2}\;$$

(4) $$\Upsilon_{\left(\mathrm{TG}\right)}=–2.027\;+\;0.003A\;+\;0.055B\;+\;0.065C\;–\;0.000009\mathrm{AB}\;–\;{0.000006A}^{2}\;–\;{0.0004B}^{2}\;–\;{0.004C}^{2}$$

where Υ_(TF)_, Υ_(TG)_, A, B, and C represented the predicted yield of TF, the yield of TG, HAc%, EtOH%, and UAE power, respectively.

#### 2.3.2. Diagnostics Plots of Model Adequacy

**Figure 2:**
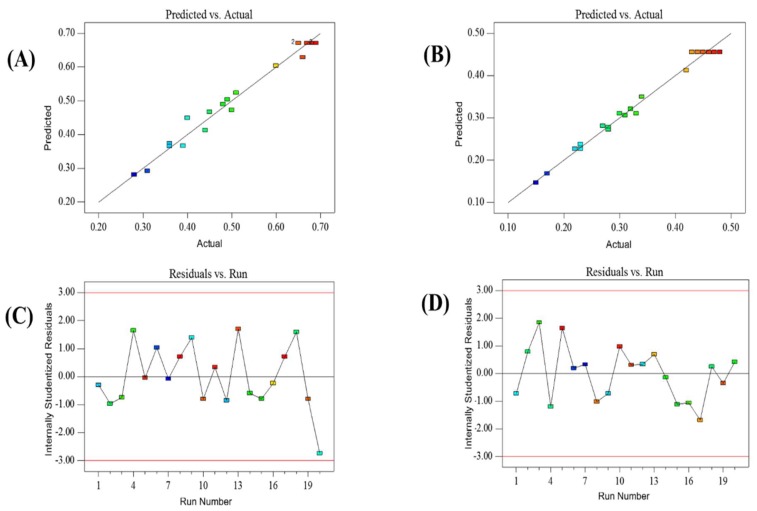
Diagnostic plots for model adequacy. (**A**) predicted versus actual of the yields of flavonoids; (**B**) predicted versus actual of the yields of ginkgolides; (**C**) internally studentized residuals of the yields of flavonoids; and, (**D**) internally studentized residuals of the yields of ginkgolides.

Two diagnostic predicted plots (versus actual and internally studentized residuals) for Υ_(TF)_ and Υ_(TG)_ were employed to detect the model adequacy. The plots of predicted versus actual for Υ_(TF)_ and Υ_(TG)_ were showed in Figure 2A,B, respectively. As shown in Figure 2A, all the points of predicted versus actual for Υ_(TF)_ were reasonably aligned, and this result indicated that the predicted values were in agreement with the experimental values. The similar results was also obtained from Figure 2B. In the plots of internally studentized residuals (Figure 2C,D), all the data points were found to lie within the limits (±3), which pointed that the models presented satisfactory fitted for Υ_(TF)_ and Υ_(TG)_. Hence, the developed models were reliable to fit the interactions between different parameters.

#### 2.3.3. Analysis of the Response Contour

**Figure 3:**
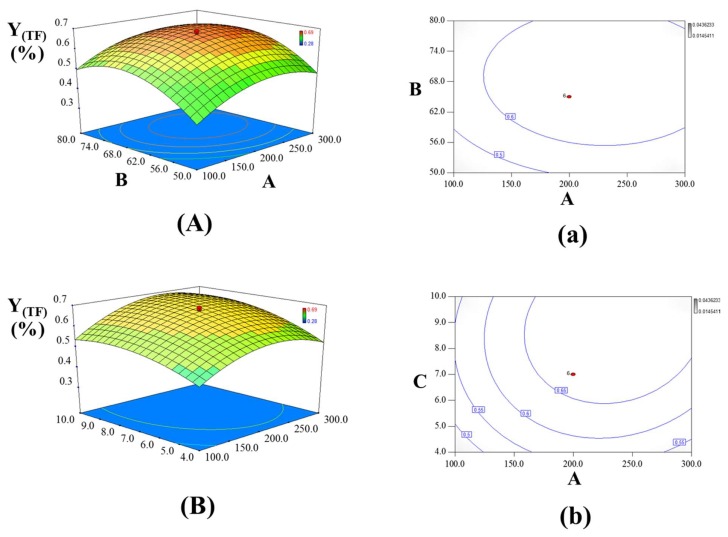

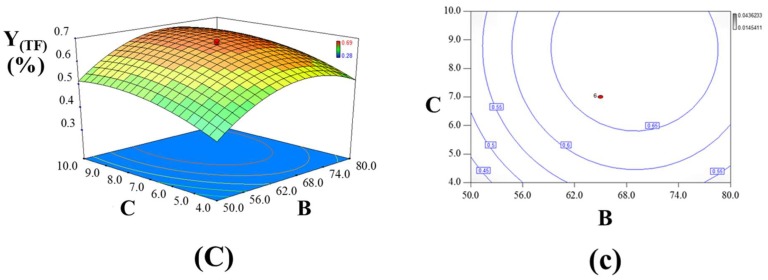
Three-dimensional contour plots showing the experimental factors and their mutual interactions: (**A**) effect of ultrasound-assisted extraction (UAE) power and EtOH% on the yield of flavonoids; (**B**) effect of UAE power and the amount of cellulase on the yield of flavonoids, and (**C**) effect of EtOH% and the amount of cellulase on the yield of flavonoids. Two-dimensional contour plots showing the experimental factors and their mutual interactions: (**a**) effect of ultrasound-assisted extraction (UAE) power and EtOH% on the yield of flavonoids; (**b**) effect of UAE power and the amount of cellulase on the yield of flavonoids, and (**c**) effect of EtOH% and the amount of cellulase on the yield of flavonoids.

**Figure 4:**
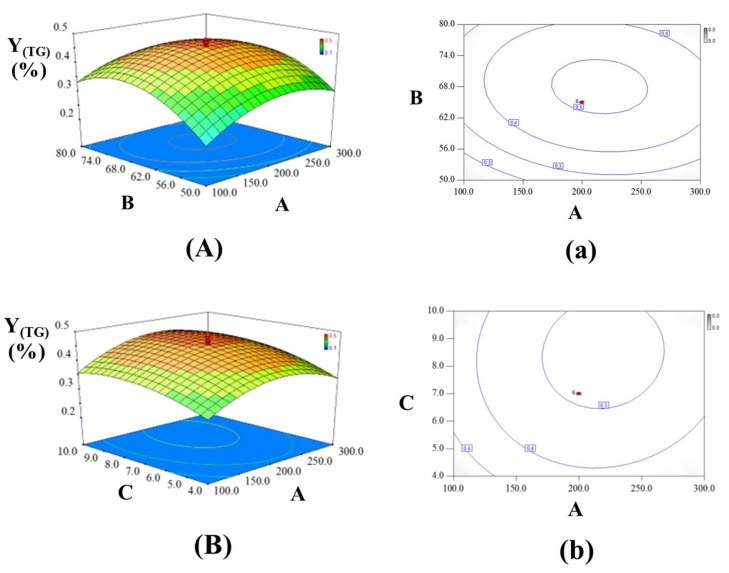

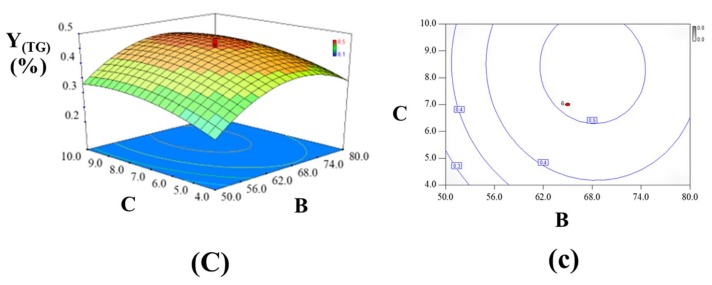
Three-dimensional contour plots showing the experimental factors and their mutual interactions: (**A**) effect of UAE power and EtOH% on the yield of ginkgolides; (**B**) effect of UAE power and the amount of cellulase on the yield of ginkgolides; and, (**C**) effect of EtOH% and UAE power on the yield of ginkgolides. Two-dimensional contour plots showing the experimental factors and their mutual interactions: (**a**) effect of UAE power and EtOH% on the yield of ginkgolides; (**b**) effect of UAE power and the amount of cellulase on the yield of ginkgolides; and, (**c**) effect of EtOH% and UAE power on the yield of ginkgolides.

Three-dimensional response surfaces were developed by the fitted Equations (3) and (4), and were plotted by the response (Z-axis), according to two factors (X and Y coordinates), holding the other one factor at zero (0-level). Drawing RSM was regarded as the best way to visualize the influence of the independent variables. The interactions between UAE power (A) and EtOH% (B) were presented in Figure 3A and Figure 4A, keeping the amount of cellulase (C) at 0 level. When the EtOH% (B) was fixed, both Υ_(TF)_ and Υ_(TG)_ rapidly increased with the increase of UAE power (A), until reaching a maximum and then rapidly decreased. From the previous reports, more bubbles were formed and collapsed with larger amplitude ultrasound waves traveling through extraction solvent under high power in UAEE process. As a result, violent shock wave and high-shear gradients might be created to cause the destruction of the cell walls. This facilitated the release of compounds significantly and enhanced the mass transfer rate simultaneously, thus leading to high yields of TF and TG from GBFL. However, the degradation or the isomerization of flavonoids and ginkgolides would occur under too high ultrasound power, which could explain the reason why the value of two responses rapidly decreased. As the EtOH% (B) increased, the yield of TF and TG increased significantly and then decreased slightly. The incomplete extraction may occur in a low EtOH% (B). In our previous reports, the contents of flavonoid aglycones were much higher than the flavonoid glycoside in GBFL [22,23]. Additionally, ginkgolides such as ginkgolide A, B, C, and bilobalide could be simultaneously detected in GBFL [22,23]. Ginkgolides and flavonoid aglycones belonged to middle-polar compounds that were easy to dissolve in relative high content of EtOH solution. Hence, higher EtOH% (B) contributed to improve dissolution of the middle-polar compounds of target compounds, resulting in the continual enhancement on the yields of TF and TG.

Figure 3B and Figure 4B showed the interaction of UAE power (A) and the amount of cellulase (C), with a fixed EtOH% (0 level). The linear and quadratic terms of UAE power (A) and the amount of cellulase (C) caused significant influences on the yields of TF and TG, however, the interactive effects between UAE power (A) and the amount of cellulase (C) showed non-significant influences. An obvious enhancement and then no significant change in the yields of TF and TG were observed with an increase in the amount of cellulase (C). The phenomenon could be explained by that the whole UAEE process needed enough cellulase to break the cell wall of certain GBFL samples for promoting penetration of extraction solvent into plant matrix and the diffusion of target compounds (flavonoids and ginkgolides) from materials to outside solvent. Therefore, a relatively larger the amount of cellulase contributed to positive influence on the yields of TF and TG. However, the constant yields of TF and TG were due to the amount of cellulase used being enough to break the cell wall of certain GBFL samples.

For the values of Υ_(TF)_ and Υ_(TG)_, EtOH% (B) and the amount of cellulase (C) contributed significant influences in both linear and quadratic manners, while the interactive effects of EtOH% (B) and the amount of cellulase (C) presented non-significant effects. The interactions between EtOH% (B) and the amount of cellulase (C) were showed in Figure 3C and Figure 4C, keeping ultrasound UAE power at 0-level. The results indicated that the effect of EtOH% (B) on both Υ_(TF)_ and Υ_(TG)_ was first increased significantly, and then decreased slightly. As the amount of cellulase (C) increased, the initial distinct increase of two responses was followed by an almost constant value.

### 2.4. Optimization and Validation of Optimized Condition

In this study, considering the fact of maximum the yields of both flavonoids and ginkgolides from GBFL, the Design–Expert 8.5 software was used for simultaneous optimization of the multiple responses by the desirability function. The desirability approach was a popular method that assigned a given score for responses and then a factor setting that maximizes the overall score would be chosen. Using Design–Expert 8.5, the optimal values of significant factors for the yields of flavonoids and ginkgolides were provided as follows: UAE power of 217.5 W, EtOH% of 67.8%, and the amount of cellulase of 8.4 mg. Furthermore, the other non-significant factors, including UAE temperature, UAE time, pH, and sample particle size were also investigated in the preliminary experiments and described in Section 2.2. Finally, for the operational convenience, the following optimum conditions were selected: UAE power of 218 W, EtOH% of 68%, the amount of cellulase of 8.4 mg, UAE temperature of 40 °C, UAE time of 20 min, pH of 5.0 and sample particle size of 40 mesh, which predicted the yields of flavonoids and ginkgolides as 0.71% and 0.46%, respectively. Under the optimum conditions, the experimental yields of flavonoids were 0.74 ± 0.05% (*n* = 3) and ginkgolides were 0.42 ± 0.06% (*n* = 3), which were very close to the predicted values, indicating that the second-order model was adequate to describe the influence of the selected UAEE variables on the extraction yields of flavonoids and ginkgolides from GBFL.

### 2.5. Experimental Design of Macroporous Resin Purification Conditions

From the above experimental design of UAEE conditions, the ideal UAEE condition was used to obtain the maximal recovery of flavonoids and ginkgolides from GBFL, but the contents of flavonoids and ginkgolides in GBFL extracts using the optimized UAEE condition still obviously failed to comply with the standardized EGb in the European, United States (U.S.), and Chinese pharmacopoeias. To overcome this problem, macroporous resin was used to further enrich the flavonoids and ginkgolides and remove the needless components from GBFL extracts.

#### 2.5.1. Selection of Macroporous Resins by Static Adsorption and Desorption

Adsorption and desorption process of macroporous resins on target constituents were not only influenced greatly by polarity and size of adsorbates, but also by dimensional structure of adsorbents, including specific surface area and pore diameter. Because middle-polar (ginkgolides and flavonoid aglycones) and polar (flavonoid glycoside) compounds were simultaneously existed in GBFL extracts, thus the type of macroporous resins used in this research were selected among weak-polar, middle-polar and polar, which were applicable to the adsorption of the target compounds. Macroporous resins (DA-201, D101, D301, HP-20, HPD400, and AB-8) were investigated and compared by the adsorption and desorption performance for flavonoids and ginkgolides. When compared to other resins, DA-201 and AB-8 presented the adsorption and desorption capacities. Although the adsorption capacity of AB-8 was not as high as DA-201, the desorption ratio of AB-8 was almost equal with DA-201. Thus, DA-201 and AB-8 were further investigated using a dynamic adsorption experiment. The result of dynamic adsorption experiment indicated that ginkgolides and flavonoids began to leak out obviously when 3 BV of the GBFL extracts were loaded on AB-8, while after 4 BV of the GBFL extracts were loaded on DA-201, the substances began to leak out apparently. Additionally, the weak-polar needless compounds began to leak out obviously when 5 BV of the GBFL extracts were loaded on AB-8, while after 6 BV of the GBFL extracts were loaded on DA-201, the weak-polar needless compounds began to leak out apparently. As the more GBFL extracts were added, the more leak on AB-8 emerged than that on DA-201. Therefore, DA-201 macroporous resin was selected for further enrich flavonoids and ginkgolides and removed the needless components from GBFL extracts.

#### 2.5.2. Effect of Ethanol Concentration on Desorption of Flavonoids and Ginkgolides

**Figure 5:**
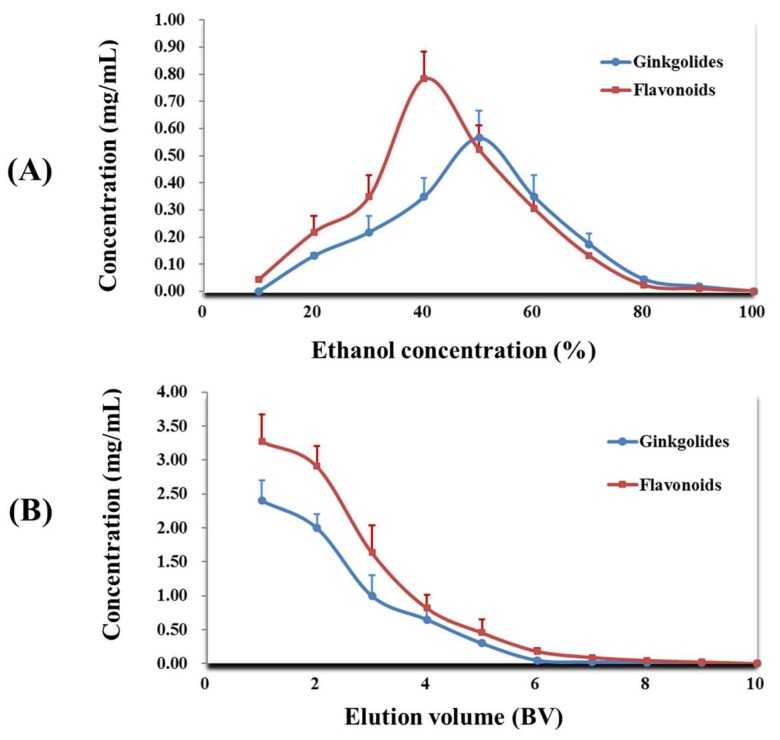
Elution conditions of flavonoids and ginkgolides from DA-201 macroporous resin. (**A**) Effect of ethanol concentration on desorption of total target compounds; and, (**B**) effect of elution volume on desorption of total target compounds. Results were expressed as the mean value ± standard deviation (*n* = 3).

To determine the proper ethanol concentration for desorption of target compounds from DA-201, continuous concentration gradient of ethanol aqueous solution was used to elute the adsorbed macroporous resin column successively. As shown in Figure 5A, the more flavonoids and ginkgolides could be cleared off when the higher the concentration of ethanol solution was employed as elution solution. More than 95% of flavonoids and ginkgolides in GBFL extracts were, respectively, eluted with 80% EtOH solution, and only 3% the weak-polar needless compounds in GBFL extracts were eluted by 80% EtOH solution. Therefore, 80% EtOH was selected as elution solution for enriching the target compounds and removing needless components from GBFL extracts.

#### 2.5.3. Effect of Elution Volume on Desorption of Flavonoids and Ginkgolides

From Figure 5B, the flavonoids and ginkgolides could be eluted partially with the first 3 BV of eluent when 80% EtOH was employed as elution solution. More than 95% of flavonoids and ginkgolides could be eluted when the eluent volume reached 5 BV. As shown in Figure 5B, only few needless components were eluted with 5 BV using 80% EtOH as the elution solution. Thus, 5 BV was selected as elution volume in this study.

Finally, the purification process of target compounds on DA-201 resin was as follows: (1) adsorption stage: sample volume was 2 mL, flow rate was 3 BV/h, operated at room temperature; (2) washing stage: DA-201 resin was washed with 2 BV of water at a 3 BV/h flow rate; and (3) desorption stage: the elution solvent was 5 BV 80% EtOH at a flow rate of 3 BV/h.

### 2.6. Evaluation of the Validated Extraction Method

This overall study aimed at maximum extracting and purifying flavonoids and ginkgolides from waste GBFL, which was highly desired for comprehensive utilization and development of *G. biloba* resources. Such extracts could be used as a good source of natural flavonoids and ginkgolides as nutraceuticals and pharmaceuticals. In this study, a simple and low-cost method of UAEE extraction, followed by macroporous resin purification was developed to achieve maximum extracting and purifying target compounds from waste GBFL and could be easily extended to industries. In the previous reports, the contents of flavonoids and ginkgolides were 0.9–2.0% and 0.3–1.1% in GBL, respectively. The contents of flavonoids and ginkgolides were 0.3–0.7% and 0.2–0.5% in GBFL, respectively. Although the contents of flavonoids and ginkgolides in raw material of GBL were obvious higher than GBFL, the percentage of total flavonoids and ginkgolides in the standardized EGb from Chinese pharmacopoeias were similar with GBFL extracts that were obtained by the optimized extraction and purification method in this study. Under the optimized extraction and purification conditions, the percentage of total flavonoids was (25.36 ± 1.03)% (*n* = 3) and ginkgolides was (12.43 ± 0.85)% (*n* = 3) from the obtained dry GBFL extracts, which was complied with the standardized EGb in Chinese pharmacopoeias. Additionally, due to the negative properties of alkylphenols, a limiting concentration of 5 μg/g for all alkylphenols was specified in the monographs for standardized EGb in the European and U.S. pharmacopoeias and a limit of 10 μg/g in the Chinese Pharmacopoeia [24]. Thus, for the quality control and safety evaluation of drug or functional foods, an efficient extraction and purification of extracts was of great importance to remove alkylphenols while retaining ginkgolides and flavonoids from *G. biloba*. In our previous reports, the contents of total alkylphenols in GBL and GBFL were 0.01–0.05% and 0.4–1.0%, respectively. For quality control of EGb, some EGb were prepared in a multi-step process for reducing alkylphenols while retaining ginkgolides and flavonoids from GBL [6,25]. However, the selective multi-step process was complex in the practical operation, even though some compounds (ginkgolides and flavonoids) were enriched, while others (alkylphenols) were removed. On the other hand, the use of the optimal multi-step process condition on an industrial scale was proved to be expensive. Due to the low contents of alkylphenols in GBFL, the process for removing alkylphenols from GBFL extracts should be more simper than GBL extracts. From the results, using the developed simple method of extraction and purification, the percentage of total alkylphenols was (0.003 ± 0.0005)% (*n* = 3) from the obtained dry GBFL extracts that were complied with the standardized EGb in European and U.S. pharmacopoeias (less than 0.005% of total alkylphenols). Therefore, this optimized process had great application potentials in the manufacturing process of natural flavonoids and ginkgolides in food and drug industries.

## 3. Conclusions

In this study, we showed that the waste GBFL could be used as a source for obtaining rich flavonoids and ginkgolides extracts using the UAEE method. UAEE extraction, followed by DA-201 macroporous resin purification, was successfully optimized for maximum retaining flavonoids and ginkgolides in GBFL extracts. Under the optimal conditions, the active and toxic components in GBFL extract can achieve the standardized EGb in China and European pharmacopoeias, respectively. Additionally, this developed process could be considered as an improvement for the flavonoids and ginkgolides extraction and purification since it allowed for the reduction of processing time and saving energy.

## 4. Experimental

### 4.1. Materials and Samples

The acetonitrile and formic acid were of HPLC grade. Deionized water was prepared from distilled water through a Milli-Q water purification system (Millipore, Bedford, MA, USA). DA-201, D101, D301, HP-20, HPD400, and AB-8 were purchased from Cangzhou Baoen, Co. Ltd (Hebei, China). Cellulase from *Trichoderma viride* (11,000 U/g) was purchased from Sigma Chemicals Co. (St. Louis, MO, USA). All of the other reagents and chemicals were of analytical grade. The standards of quercetin (**1**), apigenin (**2**), kaempferol (**3**), isorhamnetin (**4**), genkwanin (**5**), amentoflavone (**6**), bilobetin (**7**), ginkgetin (**8**), isoginkgetin (**9**), sciadopitysin (**10**), (−)-epigallocatechin (**11**), (+)-catechin hydrate (**12**), (−)-epicatechin (**13**), luteolin (**14**), kaempferol 3-*O*-α-*l*-[6‴-p-coumaroyl-(β-*d*)-glucopyranosyl-(1,2)-rhamnopyranoside] (**15**), quercetin 3-*O*-α-*l*-[6‴-p-coumaroyl-(β-*d*)-glucopyranosyl-(1,2)- rhamnopyranoside] (**16**), quercetin 3-*O*-[6-*O*-(α-*l*-rhamnosyl)-β-*d*-glucoside] (**17**), quercetin 3-*O*-β-*d*-glucoside (**18**), quercetin 3-*O*-[4-*O*-(α-*l*-rhamnosyl)-β-*d*-glucoside] (**19**), quercetin 3-*O*-α-*l*-rhamnoside (**20**), bilobalide (**21**), ginkgolide A (**22**), ginkgolide B (**23**), ginkgolide C (**24**), ginkgoneolic acid (**25**), and ginkgolic acid (**26**) were isolated from GBL in our laboratory and were identified by chemical and spectroscopic analysis. The purity of each compound was >98%.

The *G. biloba* fallen leaves were collected from Taizhou, Jiangsu, China. A voucher specimen (NJTCU 20171211) was deposited at the Herbarium in Jiangsu Collaborative Innovation Center of Chinese Medicinal Resources Industrialization, Nanjing University of Chinese Medicine, and Nanjing, China. After collection, the samples were air-dried.

### 4.2. Analytical Methods

UPLC-DAD-Q/TOF-MS was used to identify and determinate flavonoids and ginkgolides. UPLC was performed using a Waters ACQUITY UPLC system (Waters, Milford, MA, USA), equipped with a binary solvent delivery system, an autosampler, and a photodiode array (PDA) detector. An Acquity BEH C_18_ column (2.1 mm × 100 mm, 1.7 μm) that was maintained at 40 °C was used with an injection volume of 2 μL. Mobile phase A was 0.1% formic acid solution, and mobile phase B was acetonitrile solution. The flow rate was 0.4 mL/min. The linear gradient conditions were: 95–80% A (0–1.5 min), 80–70% A (1.5–7.0 min), 70–35% A (7.0–9.5 min), 35–0% A (9.5–11.0 min), and 0% A (11.0–12.5 min). The wavelength of the on-line PDA detector was set from 200 nm to 400 nm.

Mass spectrometry was carried out using a Waters Synapt mass spectrometer (Waters), equipped with an electrospray ionization source (ESI) in negative mode with scan range of *m/z* 50–1500 Da. The parameters of MS were following, as our previous report [16].

### 4.3. Extraction Procedures

The extraction process was performed with an ultrasonic device (SY-5200T, Shanghai Shenyuan Ultrasonic Instrument Co. Shanghai, China). 1 g of dried GBFL powder was mixed with 10 mL phosphate buffer (pH 3.0–6.0) and 4–10 mg cellulase. The mixture was incubated at 45 °C for 10 min in a rotary shaker at 120 rpm. Then, absolute ethanol was added to the mixture until its ethanol concentration reaching 50–80%. The reaction mixture was simultaneously irradiated at 100–300 W of UAE at the temperature of 30–80 °C for 10–30 min. After the UAE, all of the sample was, respectively, centrifuged at 13,000 rpm for 10 min to collect 3 mL of the supernatant, which were added onto the activated solid phase extraction (SPE) cartridge. After the sample had been absorbed by the cartridge, the cartridge was washed with 4 mL water, and then eluted with 4 mL methanol. The elution was then evaporated under reduced pressure at 50 °C. The residue was reconstituted in 0.3 mL of methanol with vortex. After centrifugation at 13,000 rpm for 10 min, 200 μL of supernatant was drawn and 3 μL was injected into the UPLC-DAD-Q/TOF-MS.

### 4.4. Experimental Design of UAEE Conditions

Experimental design and study were performed using a PBD, followed with a rotatable central composite design (RCCD). Initially, the PBD was applied to identify the significant variables that influenced the yields of flavonoids and ginkgolides from GBFL. Immediately, the variables of significance resulted from PBD were investigated by RCCD. The variables with confidence levels >95% were considered as significantly influencing the yields of flavonoids and ginkgolides.

#### 4.4.1. PBD

PBD was one of the widely applicable statistical experimental designs for data interpretation [26]. In this work, PBD was also performed to evaluate the significance of seven variables that were affecting the UAEE procedures, including UAE power (A); EtOH% (B); the amount of cellulase (C); UAE time (D); UAE temperature (E); pH (F); and, GBFL particle size (G). The design included 12 runs of various combinations of the independent variables coded as A–G and examined at high (+) or low (−) levels. Table 2 shows the seven factors and their levels, and the design matrix is presented in Table 3. The selected seven factors were realized with the yields of flavonoids and ginkgolides from GBFL taken as the responses. The variables were screened out using a Pareto chart, which displayed the absolute value of the effects and drew a reference line on the chart. All of the experiments were carried out in triplicate. PBD was based on the first order polynomial model shown, as standard Equation (5):

(5) $$Y_{i}=C_{0}+\;\sum_{i}C_{i}X_{i}$$

where *Y_i_* was the experimental response, *X_i_* was the independent variables, and *C*_0_ and *C_i_* were the regression coefficients for intercept and linear terms, respectively.

#### 4.4.2. RCCD

To optimize the significant independent variables from PBD, a RCCD was applied to obtain the maximum the yields of flavonoids and ginkgolides. The significant independent variables were individually modified, while the others without significance were maintained at the “0” condition. Each significant parameter was examined at five levels (−α, −1, 0, 1, +α). The data from the RCCD were analyzed by multiple regressions to fit the following quadratic equation:

(6) $$\Upsilon =\varphi_{0}+\sum_{i=1}^{n}\varphi_{i}x_{i}+\sum_{i=1}^{n}\varphi_{ii}x_{i}^{2}+\sum_{i=1}^{n-1}\sum_{j=i+1}^{n}\varphi_{ij}x_{i}x_{j}\;$$

where Υ, *φ*_0_, *φ_i_*, *φ_ii_* and *φ_ij_* indicated the response, the intercept term, the linear coefficient, the squared coefficient, and the interaction coefficient, respectively. Model adequacy was evaluated using the F ratio and the coefficient of determination (R^2^), which is represented at 5% level of significance, accordingly.

### 4.5. Experimental Design of Macroporous Resin Purification Conditions

The extract of GBFL, as obtained by the above optimal UAEE conditions, was concentrated to 2 mL under reduced pressure at 50 °C and purified using a packed macroporous resin column. The column (100 mm × 18 mm I.D.) was packed with DA-201 macroporous resin. After the loading of the sample (3 bed volume per hour, 3 BV/h), the column was washed with 3 BV (bed volume) of water, and eluted with 5 BV of 80% EtOH solution successively at a flow rate of 3 BV/h. The effluent liquids were collected and analyzed.

#### 4.5.1. Selection of Macroporous Resins by Static adsorption and Desorption

Firstly, macroporous resins such as DA-201, D101, D301, HP-20, HPD400, and AB-8 were investigated for recovery of flavonoids and ginkgolides from GBFL extracts by static adsorption. Adsorption experiments were carried out in 25 mL flasks in a water-bathing constant temperature vibrator (Shanghai Jinghong Laboratory Instrument Co., Ltd., Shanghai, China) in order to maintain temperature at 40 °C for 12 h. Solid/liquid ratio (S/L) was set at 1:4, namely 1 g of adsorbent (dry weight) for 4 mL of the above concentrated extracts of GBFL. Desorption experiments were carried out in 25 mL flasks with the same operation as adsorption, while S/L and temperature were fixed at 1:15 and 40 °C, respectively. The adsorption/desorption capacity and desorption ratio of each resin were calculated based on the previous report [15].

#### 4.5.2. Selection of Macroporous Resins by Dynamic Adsorption

DA-201 and AB-8 were tested for the adsorption capacity of flavonoids and ginkgolides from GBFL extracts by dynamic adsorption. The extracts (as described in Section 4.5) were filtered and were loaded on a packed macroporous resin column with DA-201 and AB-8, respectively (3 BV/h). The effluent liquids were collected (1 BV for each) and analyzed by UPLC-DAD-Q/TOF-MS.

#### 4.5.3. Effect of Ethanol Concentration on Desorption of Target Compounds

2 mL concentrated GBFL extracts were loaded on a column (100 mm × 18 mm I.D.) wet-packed with 5 mL of DA-201 (1 BV = 10 mL) at 3 BV/h. Then, the same column was washed with 2 BV of water (3 BV/h) and eluted with 10%, 20%, 30%, 40%, 50%, 60%, 70%, 80%, 90%, and 100% EtOH solution (5 BV for each concentration gradient, 3 BV/h) successively. In sequence, the effluent liquid of different concentration gradient was collected and analyzed.

#### 4.5.4. Effect of Elution Volume on Desorption of Target Compounds

From the result of Section 4.5.3, 80% EtOH solution was used as elution solvent. 2 mL extracts were loaded on DA-201 column (3 BV/h). After washed with 2 BV of water, the column was successively eluted with 5 BV of 80% EtOH (3 BV/h). The effluent liquid was collected (1 BV for each) and analyzed by UPLC-DAD-Q/TOF-MS.

### 4.6. Statistical Treatment of Data

Design Expert software (version 8.0, Stat-Ease, Minneapolis, MN, USA) was used for designing experiments as well as for regression analysis of the experimental data obtained.

## Figures and Tables

**Table 1:** Q/TOF–MS data obtained in electrospray ionization source (ESI^−^) mode of flavonoids, ginkgolides and alkylphenols.

No.	Identification	MS ^1^	Formula	Fragment ions (MS ^2^)
1	quercetin	301.0348 [M−H]^−^ (−2.0)	C_15_H_10_O_7_	151.0026, 107.0130, 83.0151, 65.0036
2	apigenin	269.0455 [M−H]^−^ (−1.8)	C_15_H_10_O_5_	151.0031, 8.3.0145
3	kaempferol	285.0558 [M−H]^−^ (1.9)	C_15_H_10_O_6_	255.0182, 227.0515, 151.0035
4	isorhamnetin	315.0688 [M−H]^−^ (1.3)	C_16_H_12_O_7_	285.0458, 255.0287, 151.0053
5	genkwanin	283.0622 [M−H]^−^ (4.4)	C_16_H_12_O_5_	268.0384, 240.0428, 151.0041
6	amentoflavone	537.0832 [M−H]^−^ (−3.3)	C_30_H_18_O_10_	443.0396, 375.0490, 331.1900, 203.0330
7	bilobetin	551.0980 [M−H]^−^ (−0.5)	C_31_H_20_O_10_	457.0579, 389.0671, 151.0038
8	ginkgetin	565.1128 [M−H]^−^ (−2.1)	C_32_H_22_O_10_	533.0861, 518.0638, 415.0451, 151.0035
9	isoginkgetin	565.1147 [M−H]^−^ (1.2)	C_32_H_22_O_10_	533.0852, 518.0631, 415.0444, 151.0042
10	sciadopitysin	579.1283 [M−H]^−^ (−2.3)	C_33_H_24_O_10_	533.0863, 415..0465
11	(-)-epigallocatechin	305.0780 [M−H]^−^ (1.9)	C_15_H_14_O_7_	261.0715
12	(+)-catechin hydrate	289.0677 [M−H]^−^ (2.1)	C_15_H_14_O_6_	261.0722
13	(-)-epicatechin	289.0679 [M−H]^−^ (2.5)	C_15_H_14_O_6_	261.0733
14	luteolin	285.0541 [M−H]^−^ (4.2)	C_15_H_10_O_6_	217.0512
15	kaempferol 3-*O*-α-*L*-[6‴-p-coumaroyl-(β-*d*)-glucopyranosyl-(1,2)-rhamnopyranoside]	739.2543 [M−H]^−^ (4.7)	C_33_H_40_O_19_	593.1370, 447.0830, 285.0626, 255.0439, 227.0229, 151.0048
16	quercetin 3-*O*-α-*l*-[6‴-p-coumaroyl-(β-*d*)-glucopyranosyl-(1,2)-rhamnopyranoside]	755.2302 [M−H]^−^ (2.7)	C_33_H_40_O_20_	609.1880, 463.0673, 301.0503, 149.0196
17	quercetin 3-*O*-[6-*O*-(α-*L*-rhamnosyl)-β-*d*-glucoside]	609.1853 [M−H]^−^ (3.7)	C_27_H_30_O_16_	463.0629, 301.0524, 151.0124, 149.0092
18	quercetin 3-*O*-β-*d*-glucoside	463.1170 [M−H]^−^ (2.3)	C_21_H_20_O_12_	301.0569, 149.0182
19	quercetin 3-*O*-[4-*O*-(α-*l*-rhamnosyl)-β-*d*-glucoside]	609.1848 [M−H]^−^ (2.1)	C_27_H_30_O_16_	463.0633, 301.0519, 151.0127, 149.0095
20	quercetin 3-*O*-α-*l*-rhamnoside	447.1222 [M−H]^−^ (2.5)	C_21_H_20_O_11_	301.0400, 149.0152
21	bilobalide	325.1079 [M−H]^−^ (1.6)	C_15_H_18_O_8_	281.0782, 237.1035, 251.0882, 163.1200
22	ginkgolide A	407.1463 [M−H]^−^ (2.8)	C_20_H_24_O_9_	363.1537, 351.1609, 333.1449
23	ginkgolide B	423.1521 [M−H]^−^ (4.1)	C_20_H_24_O_10_	395.1067, 367.1586
24	ginkgolide C	439.1459 [M−H]^−^ (1.9)	C_20_H_24_O_11_	411.1303, 383.1461, 365.1328, 303.1131
25	ginkgoneolic acid	319.2319 [M−H]^−^ (−2.3)	C_20_H_32_O_3_	275.2409
26	ginkgolic acid	345.2416 [M−H]^−^ (−1.8)	C_22_H_34_O_3_	301.2653

**Table 2:** Real values of the variables in the Plackett-Burman design and experimental data of the yields of total flavonoids and ginkgolides from *Ginkgo biloba* fallen leaves (*n* = 3).

Run	Factors							Responses	
	A ^a^	B	C	D	E	F	G	Y_1_^b^	Y_2_
	(W)	(%)	(mg)	(min)	(°C)		(mesh)	(%)	(%)
1	300.00	80.00	10.00	10.00	30.00	3.00	80.00	0.58	0.31
2	300.00	50.00	10.00	30.00	30.00	6.00	80.00	0.43	0.22
3	100.00	50.00	4.00	30.00	30.00	6.00	80.00	0.28	0.11
4	300.00	50.00	10.00	30.00	80.00	3.00	40.00	0.45	0.21
5	100.00	50.00	10.00	10.00	80.00	6.00	40.00	0.35	0.14
6	100.00	80.00	10.00	10.00	80.00	6.00	80.00	0.43	0.23
7	300.00	50.00	4.00	10.00	80.00	3.00	80.00	0.41	0.19
8	100.00	50.00	4.00	10.00	30.00	3.00	40.00	0.27	0.11
9	300.00	80.00	4.00	10.00	30.00	6.00	40.00	0.47	0.26
10	100.00	80.00	4.00	30.00	80.00	3.00	80.00	0.42	0.23
11	100.00	80.00	10.00	30.00	30.00	3.00	40.00	0.46	0.25
12	300.00	80.00	4.00	30.00	80.00	6.00	40.00	0.49	0.27

^a^**A**: UAE power; **B**: EtOH%; **C**: cellulose concentration; **D**: UAE time; **E**: UAE temperature; **F**: pH; **G**: GBFL particle size. ^b^**Y_1_**: The yield of total flavonoids; **Y_2_**: The yield of total ginkgolides.

**Table 3:** Analysis of variance and regression analysis of Plackett-Burman design data for the prediction of significant extraction variables.

Regression Data								
Source	Y_1_ (The Yield of Total Flavonoids)				Y_2_ (The Yield of Total Ginkgolides)			
	Effect	*F*-Value	*p*-Value	Inference	Effect	*F*-Value	*p*-Value	Inference
**Model**		33.22	0.0022	Significant		83.03	0.0004	Significant
A	0.10	91.52	0.0007	Significant	0.065	169.00	0.0002	Significant
B	0.11	103.71	0.0005	Significant	0.095	361.00	<0.0001	Significant
C	0.06	30.86	0.0051	Significant	0.032	40.11	0.0032	Significant
D	0.003	0.095	0.7730		0.008	2.78	0.1709	
E	0.01	0.86	0.4069		0.002	0.11	0.7556	
F	−0.023	4.67	0.0969		−0.012	5.44	0.0800	
G	0.01	0.86	0.4069		0.008	2.78	0.1709	

**Table 4:** Rotatable central composite design (RCCD) with experimental values for the yields of total flavonoids and ginkgolides from *Ginkgo biloba* fallen leaves, analysis of variance (ANOVA) for response surface quadratic model, and fit statistics for the response values (*n* = 3).

CCD Experiments						Analysis of Variance (ANOVA)				
Run	A ^a^	B	C	Y_1_^b^	Y_2_	Source	Y_1_		Y_2_	
	(W)	(%)	(mg)	(%)	(%)		*F*-Value	*p*-Value	*F*-Value	*p*-Value
**1**	31.82	65.00	7.00	0.36	0.23	Model	46.61	<0.0001	82.03	<0.0001
**2**	200.00	90.23	7.00	0.45	0.28	A	35.71	0.0001	23.91	0.0006
**3**	368.18	65.00	7.00	0.51	0.33	B	49.47	<0.0001	69.56	<0.0001
**4**	300.00	80.00	4.00	0.5	0.27	C	45.79	<0.0001	50.88	<0.0001
**5**	200.00	65.00	7.00	0.67	0.48	AB	0.13	0.7229	5.55	0.0402
**6**	100.00	50.00	4.00	0.31	0.17	AC	1.79	0.2108	1.15	0.3092
**7**	200.00	39.77	7.00	0.28	0.15	BC	0.015	0.9056	0.41	0.5349
**8**	200.00	65.00	7.00	0.69	0.44	A^2^	109.42	<0.0001	216.92	<0.0001
**9**	300.00	50.00	4.00	0.39	0.22	B^2^	187.44	<0.0001	400.59	<0.0001
**10**	200.00	65.00	7.00	0.65	0.47	C^2^	36.92	0.0001	61.08	<0.0001
**11**	200.00	65.00	7.00	0.68	0.46	Lack of fit	4.02	0.0763	0.56	0.7323
**12**	100.00	50.00	10.00	0.36	0.23					
**13**	200.00	65.00	12.05	0.66	0.42					
**14**	100.00	80.00	10.00	0.48	0.32					
**15**	300.00	50.00	10.00	0.49	0.3	**Credibility Analysis of the Regression Equations**				
**16**	300.00	80.00	10.00	0.6	0.34					
**17**	200.00	65.00	7.00	0.69	0.43	**Index** **Mark**	**CV%**	**R^2^**	**Adj. R^2^**	**Pre. R^2^**
**18**	100.00	80.00	4.00	0.44	0.28					
**19**	200.00	65.00	7.00	0.65	0.45	**Y_1_**	5.67	0.9767	0.9558	0.8517
**20**	200.00	65.00	1.95	0.4	0.31	**Y_2_**	5.02	0.9866	0.9746	0.9513

^a^**A**: UAE power; **B**: EtOH%; **C**: cellulose concentration. ^b^**Y_1_**: The yield of total flavonoids; **Y_2_**: The yield of total ginkgolides.
